# Patient Self‐Reported Global Satisfaction With Rivaroxaban in Patients With Atrial Fibrillation: A Comparison Between Oral Anticoagulation‐Naïve Versus Oral Anticoagulation‐Experienced Patients

**DOI:** 10.1002/clc.70461

**Published:** 2026-09-02

**Authors:** Bogdan G. Markovic, Miroslav Mihajlovic, Aleksandar Kocijancic, Jelena Simic, Milan Marinkovic, Vladan Kovacevic, Milica Ivanisevic, Nikola Isailovic, Nebojsa Mujovic, Belma Pojskic, Tatjana Potpara, the GUARDIAN Investigators

**Affiliations:** ^1^ Medical Faculty University of Belgrade Belgrade Serbia; ^2^ Cardiology Clinic, University Clinical Centre of Serbia Belgrade Serbia; ^3^ Department of General Internal medicine Zenica Cantonal Hospital, Faculty of Medicine, University of Zenica Zenica Bosnia and Herzegovina

**Keywords:** atrial fibrillation, OAC‐naïve patients, patient self‐reported satisfaction, patient‐reported outcome measures, rivaroxaban

## Abstract

**Background:**

Long‐term oral anticoagulant therapy (OAC) is essential to effective stroke prevention in patients with atrial fibrillation (AF), but adherence to OAC is a concern. Patient self‐reported satisfaction with OAC is a major contributor to OAC adherence. We compared the levels of and time trends in patient global satisfaction with rivaroxaban in OAC‐naïve and OAC‐experienced AF patients.

**Methods:**

An international, prospective, observational study of treatment with generic rivaroxaban included direct oral anticoagulant (DOAC)‐eligible AF patients, whether OAC‐naïve or previously taking any OAC except rivaroxaban. At enrollment, all participants were prescribed rivaroxaban. Patient self‐reported global satisfaction with rivaroxaban was assessed at 3, 6, and 12 months using a modified Treatment Satisfaction Questionnaire for Medication Version 2 (TSQM‐II). The lower the TSQM‐II value, the higher patient satisfaction level.

**Results:**

Of 1020 participants (mean age 68.02 ± 9.65 y, 43.6% female), 630 (61.8%) were OAC‐naïve. The mean patient satisfaction level was consistently higher in OAC‐experienced versus OAC‐naïve patients across all follow‐up visits (1.41 ± 0.518 vs. 1.49 ± 0.604, *p* = 0.034, 1.45 ± 0.552 vs. 1.56 ± 0.585, *p* = 0.004 and 1.40 ± 0.511 vs. 1.52 ± 0.540, *p* < 0.001 at 3, 6, and 12 months, respectively). OAC‐naïve status was consistently significantly associated with the level of patient satisfaction during follow‐up, and significant fluctuations in patient satisfaction levels were observed at 6 months among OAC‐naïve but not OAC‐experienced patients.

**Conclusion:**

Our results suggest that OAC‐naïve patients need additional support, reassurance and more intense regular clinical follow‐up, particularly in the first 6 months after initiation of DOAC therapy.

## Graphical abstract

Comparisons of the levels of and time trends in patient global satisfaction with rivaroxaban in OAC‐naïve and OAC‐experienced AF patients using a 5‐point scale. Our results suggest that OAC‐naïve patients need additional support, reassurance and more intense clinical follow‐up, particularly in the first 6 months after initiation of DOAC therapy.

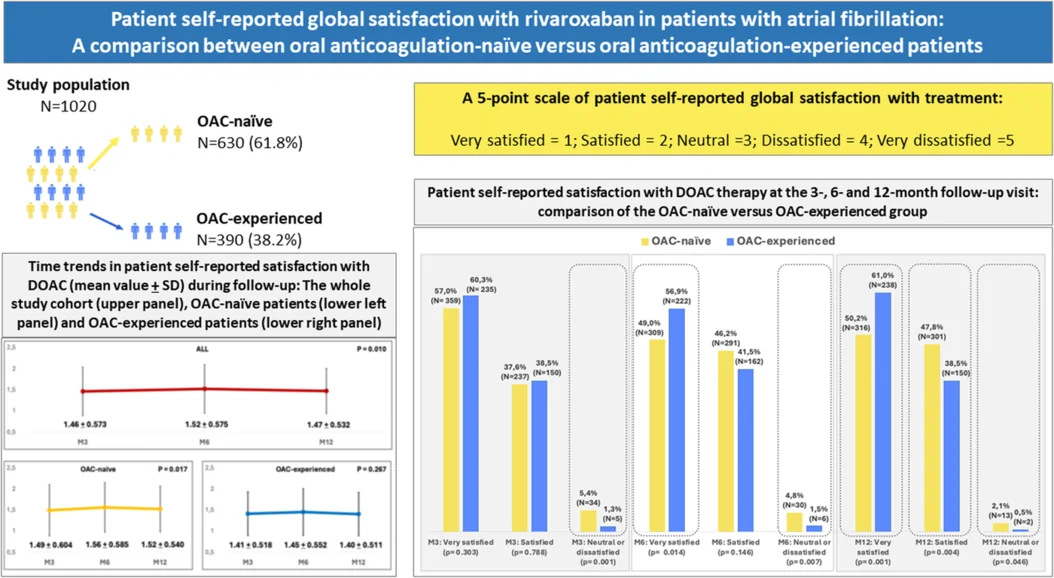

## 1 Introduction

Long‐term oral anticoagulant therapy (OAC) is a cornerstone of effective prevention of thromboembolic events in patients with atrial fibrillation (AF) who are at increased risk of stroke [1]. Following landmark randomized clinical trials (RCTs), which have shown that direct oral anticoagulants (DOACs) were at least as effective as Vitamin K antagonists (VKAs) but more convenient for long‐term use and safer regarding the risk of major (especially intracranial) bleeding [2], DOACs have largely prevailed over VKAs among DOAC‐eligible patients with AF using OAC [3].

Along with the increasing use of OAC, the implementation of a structured integrated care for patients with AF (including risk factor and comorbidity management, as well as rhythm control strategies) is also increasing [4]. Notwithstanding the resultant overall improvement in effective prevention of AF‐related stroke, several main concerns still need attention in routine clinical practice, including the problem of non‐adherence or suboptimal adherence to long‐term OAC [5]. Indeed, recent reports suggest that approximately one in seven OAC‐eligible AF patients at increased risk of stroke refuse long‐term OAC therapy, whereas in one of three to four patients taking OAC adherence to therapy would be suboptimal in the long term [5]. In patients with AF, poor adherence to OAC is associated with increased risk of major adverse clinical outcomes such as stroke, bleeding, hospitalization and death, as well as increased economic costs [6].

Multiple determinants of non‐adherence or suboptimal adherence to therapy have been identified, including medication‐, medical condition‐, socioeconomic‐, healthcare system‐ and patient‐related factors [5]. In patients with AF, patient‐reported satisfaction with OAC is shown to be an important determinant of adherence to OAC therapy [7]. In one study, for example, a lower level of patient satisfaction was significantly associated with a threefold increase in the incidence of stroke [8].

Several studies have investigated patient satisfaction with OAC therapy in patients with AF, many comparing DOACs and VKAs [6, 8, 9, 10, 11, 12, 13, 14, 15, 16, 17, 18]. While mostly limited by a small sample size, retrospective design and/or the use of unvalidated instruments, these studies consistently showed a relatively high level of patient satisfaction with OAC therapy, with no or minimal difference between DOACs and VKAs [19, 20]. In addition, several studies reported higher levels of patient satisfaction with rivaroxaban compared with other DOACs, most likely owing to its convenience [11, 21]. However, whether there are differences in the level of satisfaction with OAC therapy between OAC‐naïve compared with OAC‐experienced patients with AF is largely unknown. Notably, OAC‐naïve patients represent a vulnerable AF population at increased risk of major adverse cardiovascular outcomes early in the course of treatment with gradual “stabilization” over time [22]. Whereas the “early” risk of bleeding in these patients is related to initiation of OAC, the increased risk of thromboembolic events is largely mediated by “early” non‐adherence or suboptimal adherence to OAC therapy [23].

In line with earlier studies, we have also reported previously a persistently high overall level of patient global satisfaction with DOAC therapy (rivaroxaban) over a 12‐month follow‐up in the Guardian study [24]. In the present prespecified analysis of the Guardian study, we explored the hypothesis that patient self‐reported satisfaction with rivaroxaban and time trends in patient satisfaction may significantly differ in OAC‐naïve and OAC‐experienced patients with AF.

## 2 Materials and Methods

The Guardian study protocol has been previously described in detail [24]. Briefly, the Guardian study was an international, non‐interventional, prospective observational 1‐year follow‐up study of generic rivaroxaban Xerdoxo (Krka, d. d., Novo mesto, Slovenia) 20 mg once daily (or 15 mg once daily, as indicated) in adult patients (age > 18 years) with confirmed diagnosis of AF in whom long‐term OAC was indicated for stroke prevention and who were DOAC‐eligible with no contraindication to OAC therapy. Patients were enrolled whether OAC‐naïve (i.e., not taking OAC before) or already taking any OAC except rivaroxaban. The study was conducted in 14 hospital‐based centers in the Republic of Serbia and Bosnia and Herzegovina. At enrollment, all participants provided a written informed consent, and the study was approved by respective Institutional Review Board in each participating center.

Of 1092 patients enrolled in the main Guardian study, generic rivaroxaban was permanently discontinued before the 12‐month follow‐up visit in 72 patients (6.6%) owing to reasons such as the occurrence of contraindication to further OAC therapy (three patients), death (eight patients), unacceptable adverse effects (15 patients), unaffordable rivaroxaban price (three patients), patient's decision to stop the treatment (15 patients), physician's decision (11 patients) or unknown reasons (17 patients), and these patients were not included in the present main analysis.

### 2.1 Data Collection

Data were collected using an electronic case report form (eCRF) at study enrollment (baseline) and follow‐up visits at 3, 6, and 12 months.

At study enrollment, data on patient's demographics, cardiovascular risk factors, stroke and bleeding risk, medical history and previous and concomitant treatment were collected.

During follow‐up, information about treatment with generic rivaroxaban and study primary endpoints (i.e., thromboembolic events, major and non‐major bleeding events) and secondary endpoints (i.e., adverse events and patient's and investigator's satisfaction with generic rivaroxaban therapy) was collected at the 3‐, 6‐, and 12‐month follow‐up visit.

### 2.2 Outcome Measures

Patient self‐reported global satisfaction with generic rivaroxaban treatment was assessed using a modified Treatment Satisfaction Questionnaire for Medication Version 2 (TSQM‐II) [25] section referring to global satisfaction with the medication. The original TSQM‐II tool has four domains (Effectiveness, Side effects, Convenience and Global satisfaction) with responses collected via Likert‐type scales of 5 or 7 points or dichotomous (Yes/No) and licensing is required for its use. The TSQM‐II Global satisfaction domain explores the patient's overall appreciation of the treatment's performance, measured using a 7‐point scale with responses ranging from extremely satisfied to extremely dissatisfied. A validated translation of the original TSQM‐II questionnaire to Croatian language is available in Bosnia and Herzegovina (www.iqvia.com). In the present study, patient self‐reported global satisfaction with generic rivaroxaban was measured using a single question and a modified 5‐point scale (i.e., Very satisfied [1 point], Satisfied [2 points], Neither satisfied nor dissatisfied [3 points], Dissatisfied [4 points] and Very dissatisfied [5 points]). Hence, the higher the scale value, the lower the patient self‐reported global satisfaction with DOAC. The 5‐point scale was derived via an informal assessment among 35 patients with AF taking rivaroxaban.

Data collection and analyses were restricted to the global satisfaction assessment only, since all study participants were taking rivaroxaban during the whole study period, no comparison among different OAC agents was possible, and no pre‐planned intervention aiming to improve adherence to treatment was undertaken during the study period.

### 2.3 Statistical Analyses

Data describing the study cohort are presented as number and percentage for categorical variables and mean ± standard deviation and/or median with interquartile range (IQR) for continuous variables.

The prespecified primary analyses, that is comparisons between the OAC‐naïve and OAC‐experienced study group, were made using Student's t‐test, Mann‐Whitney U test or Chi‐square test, as appropriate.

Exploratory analyses included univariate logistic regression method which was used to assess the relationship of independent variables (patient demographics, cardiovascular risk factors, stroke and bleeding risk, medical history and previous treatment with OAC) shown in Table 1 with patient self‐reported global satisfaction with generic rivaroxaban therapy at the 3‐, 6‐, and 12‐month follow‐up visit. The patient satisfaction levels “Very satisfied” and “Satisfied” were entered into analyses as separate dependent variables, while the levels “Neither satisfied nor dissatisfied,” “Dissatisfied” and “Very dissatisfied” were summed up into a single variable named “Neutral or dissatisfied” which was then managed as a single dependent variable. This was done because of the very small number of dissatisfied and very dissatisfied patients at all follow‐up visits (i.e., one patient [0.1%] per each visit).

**TABLE 1:** Demographic data and study cohort characteristics at enrollment (overall and comparison between OAC‐naïve and OAC‐experienced patients).

Variable	All *N* = 1020 (100%)	OAC‐naïve *N* = 630 (61.8%)	OAC‐experienced *N* = 390 (38.2%)	*p* value
Age (years)	68.02 ± 9.65 (range 31–90)	67.25 ± 10.10 (range 31–90)	69.25 ± 8.74 (range 39–88)	0.001
Age under 65 years	308 (30.2)	210 (33.3)	98 (25.1)	0.006
Age 65‐74 years	460 (45.1)	270 (42.9)	190 (48.7)	0.068
Age 75‐80 years	173 (17.0)	103 (16.3)	70 (17.9)	0.508
Age over 80 years	79 (7.7)	47 (7.5)	32 (8.2)	0.665
Female sex	445 (43.6)	274 (43.5)	171 (43.8)	0.912
First‐diagnosed AF	430 (42.2)	416 (66.0)	14 (3.6)	< 0.001
CHA_2_DS_2_‐VASc score	3.16 ± 1.54 (range 0–9)	3.04 ± 1.52 (range 0–9)	3.36 ± 1.56 (range 0–8)	0.001
HAS‐BLED score	1.19 ± 0.92 (range 0–6)	1.10 ± 0.84 (range 0–4)	1.33 ± 1.02 (range 0–6)	< 0.001
Body Mass Index	27.66 ± 3.74 (range 20–50)	27.67 ± 3.80 (range 20–50)	27.65 ± 3.65 (range 20–48)	0.954
Overweight or obese	785 (77.0)	481 (76.3)	304 (77.9)	0.555
Current smoker	183 (17.9)	122 (19.4)	61 (15.6)	0.132
Hyperlipidaemia	502 (49.2)	278 (44.1)	224 (57.4)	< 0.001
Any comorbidity	976 (95.7)	592 (94.0)	384 (98.5)	< 0.001
Hypertension	903 (88.5)	553 (87.8)	350 (89.7)	0.338
Coronary artery disease	232 (22.7)	130 (20.6)	102 (26.2)	0.041
Diabetes mellitus	232 (22.7)	133 (21.1)	99 (25.4)	0.114
Heart failure	262 (25.7)	149 (23.7)	113 (29.0)	0.059
Prior stroke/TIA	89 (8.7)	48 (7.6)	41 (10.5)	0.112
Renal impairment	77 (7.5)	38 (6.0)	39 (10.0)	0.020
Obstructive sleep apnea	6 (0.6)	4 (0.6)	2 (0.5)	1.000
Multimorbidity	736 (72.2)	434 (68.9)	302 (77.4)	0.003

*Note:* The CHA_2_DS_2_–VASc stroke risk score (Congestive heart failure, Hypertension, Age > 75 years, Diabetes mellitus, Stroke, Vascular disease, Age 65–74 years, Sex category) was calculated for stroke risk assessment; the HAS‐BLED bleeding risk score (Hypertension [uncontrolled], Abnormal renal and/or liver function, Stroke history, Bleeding history, Labile International Normalized Ratio, Elderly [> 65 years], Drugs [non‐steroidal anti‐inflammatory drugs – NSAIDs, aspirin]/Alcohol use [> 8 drinks per week]).

Abbreviations: AF, atrial fibrillation; OAC, oral anticoagulant therapy; TIA, transient ischemic attack.

Furthermore, the variables statistically significantly associated with a given dependent variable on univariate logistic regression analysis were subsequently entered into the respective multivariate logistic regression model, adjusted for age, sex, CHA2DS2‐VASc score, HAS‐BLED score, Multimorbidity, Comorbidity, Renal Impairment, Hyperlipidaemia and First‐diagnosed AF. Before proceeding with multivariate logistic regression analyses, the collinearity between the variables of interest (i.e., first‐diagnosed AF and OAC‐naïve status) was tested using a two‐step approach. First, the Pearson Chi‐square test and Phi coefficient were calculated, and then Variance Inflation Factors (VIF) and tolerance values were calculated. A VIF of less than 2.5 and tolerance above 0.4, as well as a Phi coefficient of less than 0.7 were considered as an acceptable level of independence.

In addition to considering specific levels of patient satisfaction as dichotomous variables, we have also conducted exploratory univariate and multivariate linear regression analyses with dependent variables being Patient satisfaction at 3‐, 6‐, and 12‐month follow‐up visits as continuous variables. The independent variables were the same as for logistic regression analyses, and the adjustments of multivariate models was performed using the same list of variables as for logistic regression analyses.

The two‐sided *P* values of < 0.05 were considered statistically significant.

Time trends in patient self‐reported satisfaction with DOAC during follow‐up were assessed using the Friedman test, since the Shapiro‐Wilk test showed that patient satisfaction data does not follow a normal distribution. Changes in the proportion of patients with specific levels of patient‐reported satisfaction with DOAC within the OAC‐naïve and OAC‐experienced group at follow‐up visits were assessed using the non‐parametric McNemar's test to determine the significance of observed shifts in proportions between two related groups.

All the analyses were done on observed data only. No methods for the imputation of missing data were used. Data were analyzed using SPSS 31.0.0.0.

### 2.4 Sensitivity Analyses

To address possible selection bias, we have conducted sensitivity analyses in the whole study cohort (*n* = 1092), including 72 patients in whom generic rivaroxaban had been permanently discontinued at any time point before the 12‐month follow‐up visit.

## 3 Results

Baseline characteristics of the present sub‐study cohort are shown in Table 1.

Of 1020 patients (mean age 68.02 ± 9.65 years, 43.6% female), 630 patients (61.8%) were OAC‐naïve. These patients were significantly younger, with lower mean stroke and bleeding risk and more often had a first‐diagnosed AF compared with OAC‐experienced patients (Table 1). Of 390 OAC‐experienced patients, 260 patients (66.7%) were previously taking a VKA, 124 (31.8%) were taking a DOAC and 6 patients (1.5%) were on a low molecular weight heparin (LMWH).

Overall, the most prevalent cardiovascular risk factors were an increased Body Mass Index and hyperlipidaemia, and the latter was significantly more frequent among OAC‐experienced than in OAC‐naive patients. Most patients (95.7%) had at least one comorbidity (most commonly hypertension), and 72.2% of patients were multimorbid (Table 1). Comorbidity and multimorbidity were present significantly less often in OAC‐naive compared with OAC‐experienced patients (both *p* < 0.005).

### 3.1 Patient Self‐Reported Satisfaction With DOAC Therapy

Overall, patient self‐reported global satisfaction with generic rivaroxaban remained high across all follow‐up visits. The mean patient satisfaction values (the lower the value, the higher the level of satisfaction) were 1.46 ± 0.573 at 3 months, 1.52 ± 0.575 at 6 months and 1.47 ± 0.532 at 12 months. The median value of patient satisfaction was 1 (IQR of 1–2) across all three study visits. The level of patient satisfaction was consistently higher in OAC‐experienced compared with OAC‐naïve patients at each follow‐up visit, as assessed comparing the respective mean values (1.41 ± 0.518 vs. 1.49 ± 0.604, *p* = 0.034 at 3 months, 1.45 ± 0.552 vs. 1.56 ± 0.585, *p* = 0.004 at 6 months and 1.40 ± 0.511 vs. 1.52 ± 0.540, *p* < 0.001 at 12 months). The median value of patient satisfaction was 1 (IQR 1–2) in both the OAC‐naïve and the OAC‐experienced groups at all follow‐up visits, excluding the 6‐month follow‐up visit for the OAC‐naïve group, where it was 2 (IQR 1–2).

**FIGURE 1:**
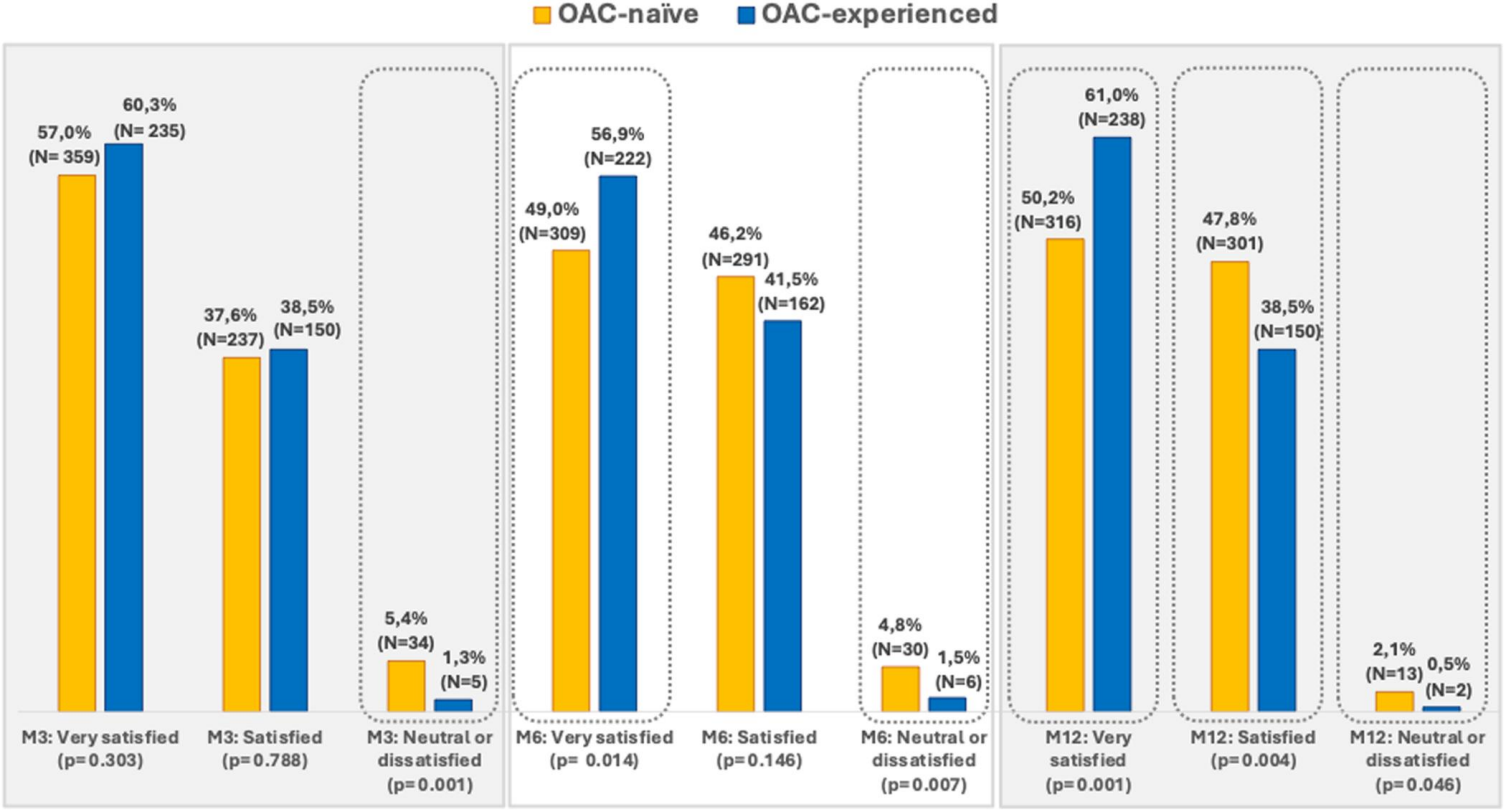
Patient self‐reported satisfaction with DOAC therapy at the 3‐, 6‐, and 12‐month follow‐up visit: comparison of the OAC‐naïve versus OAC‐experienced group. N, number; M, month; OAC, oral anticoagulant therapy. Dotted line rectangles mark the differences with statistical significance (*p* < 0.05).

Several statistically significant differences between the OAC‐naïve and OAC‐experienced group were observed throughout the study (Figure 1).

At the 3‐month follow‐up visit, a total of 594 patients (58.2%) were very satisfied, 387 (37.9%) were satisfied, and 39 patients (3.8%) were neutral or dissatisfied. While the proportions of very satisfied and satisfied patients were comparable in the OAC‐naïve and OAC‐experienced group, a significantly greater proportion of OAC‐naïve patients was neutral or dissatisfied (Figure 1, left panel).

At the 6‐month follow‐up visit, a total of 531 patients (52.0%) were very satisfied, 453 (44.4%) were satisfied, and 36 patients (3.5%) were neutral or dissatisfied. In comparison to the OAC‐experienced group, OAC‐naïve patients were less often very satisfied and more often dissatisfied (both *p* < 0.05, Figure 1, middle panel).

At the 12‐month follow‐up visit, a total of 554 patients (54.3%) were very satisfied, 451 (44.2%) were satisfied, and 15 patients (1.5%) were neutral or dissatisfied. Compared with the OAC‐experienced group, OAC‐naïve patients were less often very satisfied, and more often satisfied or neutral/dissatisfied (all *p* < 0.05, Figure 1, right panel).

Exploratory univariate logistic regression analyses with statistical significance and multivariate logistic regression models are shown in Table 2. The whole set of univariate analyses is provided in the Supporting file (Supporting Information S1: Tables 4–12).

**TABLE 2:** Univariate and multivariate logistic regression analyses of patient self‐reported satisfaction with DOAC therapy at the 3‐, 6‐, and 12‐month follow‐up visit.

	Univariate logistic regression			Multivariate logistic regression^a^		
M3: Very satisfied						
* Variable*	OR	95% CI	*p* value	OR	95% CI	*p* value
Age over 80	0.58	0.36–0.91	0.019	0.62	0.39–0.99	0.045
Hyperlipidaemia	1.73	1.34–2.22	< 0.001	1.81	1.40–2.34	< 0.001
Multimorbidity	1.33	1.01–1.76	0.042			
OAC‐naive	0.87	0.68–1.13	0.303			
OAC‐experienced	1.14	0.89–1.48	0.303			
M3: Satisfied						
Age over 80	1.76	1.11–2.78	0.017	1.65	1.04–2.62	0.035
Hyperlipidaemia	0.68	0.52–0.87	0.003	0.65	0.50–0.84	0.001
OAC‐naive	0.97	0.74–1.25	0.788			
OAC‐experienced	1.04	0.80–1.34	0.788			
M3: Neutral or dissatisfied						
First‐diagnosed AF	2.54	1.30–4.95	0.006			
Hyperlipidaemia	0.297	0.14–0.63	0.002	0.34	0.16–0.72	0.005
OAC‐naive	4.39	1.70–11.33	0.002	3.88	1.50–10.04	0.005
OAC‐experienced	0.23	0.09–0.59	0.002			
M6: Very satisfied						
Hyperlipidaemia	1.41	1.10–1.80	0.007	1.38	1.07–1.77	0.012
OAC‐naive	0.73	0.57–0.94	0.015	0.74	0.57–0.95	0.020
OAC‐experienced	1.37	1.07–1.77	0.015			
M6: Satisfied						
Age	1.02	1.01–1.03	0.006	1.02	1.01–1.03	0.004
Age over 80	1.83	1.15–2.91	0.011			
Hyperlipidaemia	0.75	0.59–0.96	0.024	0.74	0.58–0.95	0.017
OAC‐naive	1.21	0.94–1.56	0.146			
OAC‐experienced	0.83	0.64–1.07	0.146			
M6: Neutral or dissatisfied						
Age	0.96	0.94–0.99	0.021	0.95	0.91–0.98	0.002
First‐diagnosed AF	2.85	1.41–5.76	0.004			
OAC‐naive	3.20	1.32–7.76	0.010	3.33	1.34–8.25	0.009
OAC‐experienced	0.31	0.13–0.76	0.010			
M12: Very satisfied						
First‐diagnosed AF	0.73	0.57–0.94	0.013			
Body Mass Index	1.04	1.01–1.07	0.040			
Current smoker	0.70	0.51–0.96	0.029	0.67	0.48–0.94	0.019
Hyperlipidaemia	1.57	1.22–2.01	< 0.001	1.55	1.20–2.00	0.001
Renal impairment	1.87	1.12–2.99	0.017	1.86	1.11–3.11	0.018
OAC‐naive	0.64	0.50–0.83	0.001	0.67	0.52–0.88	0.003
OAC‐experienced	1.56	1.20–2.01	0.001			
M12: Satisfied						
First‐diagnosed AF	1.30	1.01–1.66	0.043			
Body Mass Index	0.96	0.93–0.99	0.025			
Current smoker	1.42	1.03–1.96	0.032	1.54	1.10–2.15	0.012
Hyperlipidaemia	0.62	0.48–0.80	< 0.001	0.65	0.51–0.84	0.001
Renal impairment	0.58	0.36–0.96	0.033	0.59	0.35–0.98	0.042
OAC‐naive	1.46	1.13–1.89	0.004	1.39	1.07–1.81	0.015
OAC‐experienced	0.68	0.53–0.88	0.004			
M12: Neutral or dissatisfied						
OAC‐naive	4.09	0.92–18.21	0.065			
OAC‐experienced	0.25	0.06–1.09	0.065			

Abbrevations: AF, atrial fibrillation; CI, Confidence Interval; M, month; OAC, oral anticoagulant therapy; OR, Odds Ratio.

^a^ adjusted for Age, Sex, CHA2DS2‐VASc score, HAS‐BLED score, Multimorbidity, Comorbidity, Renal Impairment, Hyperlipidaemia and First‐diagnosed AF.

On univariate analyses, OAC‐naïve patients had significantly greater odds of being neutral to (or dissatisfied with) DOAC therapy at the 3‐ and 6‐month follow‐up visits, and 46% greater odds of being satisfied with DOAC at the 12‐month follow‐up visit relative to OAC‐experienced patients, whereas OAC‐experienced patients had significantly greater odds of being very satisfied with DOAC at the 6‐ and 12‐month follow‐up visits (Table 2).

On exploratory multivariate analyses, OAC‐naïve patients had 3.9‐fold greater odds of being neutral to (or dissatisfied with) DOAC therapy at the 3‐ month follow‐up visit and 26% lower odds of being very satisfied with OAC therapy at the 6‐month follow‐up visit (Table 2). At the 12‐month follow‐up visit, OAC‐naïve patients had 33% lower odds of being very satisfied but 39% greater odds of being satisfied with DOAC therapy relative to OAC‐experienced patients (Table 2).

Collinearity testing for First‐diagnosed AF and OAC‐naïve status has shown a significant relationship between these two variables (Chi‐square *p* value < 0.001). Nevertheless, since VIF of 1.6, tolerance of 0.622 and Phi coefficient of 0.614 show acceptable levels of independence, the variable First‐diagnosed AF was retained in multivariate analyses.

Univariable and multivariate linear regression analyses of patient satisfaction at the 3‐, 6‐ and 12‐month follow‐up visits yielded broadly comparable results as logistic regression analyses (see Supporting Information S1: Tables 13–16).

### 3.2 Patient Satisfaction With DOAC Therapy in OAC‐Experienced Patients per Prior OAC Type

A comparison of patient satisfaction with generic rivaroxaban therapy between patients previously taking a VKA versus those taking a non‐VKA OAC agent revealed no statistically significant differences at any follow‐up visit (Table 3). Of note, among 130 non‐VKA patients, 81 patients were taking dabigatran, 43 patients were taking apixaban and six patients were receiving LMWH.

**TABLE 3:** Patient self‐reported satisfaction with DOAC therapy at the 3‐, 6‐, and 12‐month follow‐up visit: comparison among the OAC‐experienced group.

Variable	All OAC experienced patients *N* = 390 (100%)	VKA *N* = 260 (66.7%)	Non‐VKA *N* = 130 (33.3%)	*p* value
M3: Very Satisfied	235 (60.3)	162 (62.3)	73 (56.2)	0.242
M3: Satisfied	150 (38.5)	96 (36.9)	54 (41.5)	0.377
M3: Neutral or dissatisfied	5 (1.3)	2 (0.8)	3 (2.3)	0.339
M6: Very satisfied	222 (56.9)	151 (58.1)	71 (54.6)	0.515
M6: Satisfied	162 (41.5)	105 (40.4)	57 (43.8)	0.513
M6: Neutral or dissatisfied	6 (1.5)	4 (1.5)	2 (1.5)	1.000
M12: Very satisfied	238 (61.0)	161 (61.9)	77 (59.2)	0.607
M12: Satisfied	150 (38.5)	99 (38.1)	51 (39.2)	0.825
M12: Neutral or dissatisfied	2 (0.5)	0 (0.0)	2 (1.5)	0.111

Abbreviations: M, month; N, number; OAC, oral anticoagulant therapy; VKA, vitamin K antagonist.

### 3.3 Time Trends in Patient Self‐Reported Satisfaction With DOAC

**FIGURE 2:**
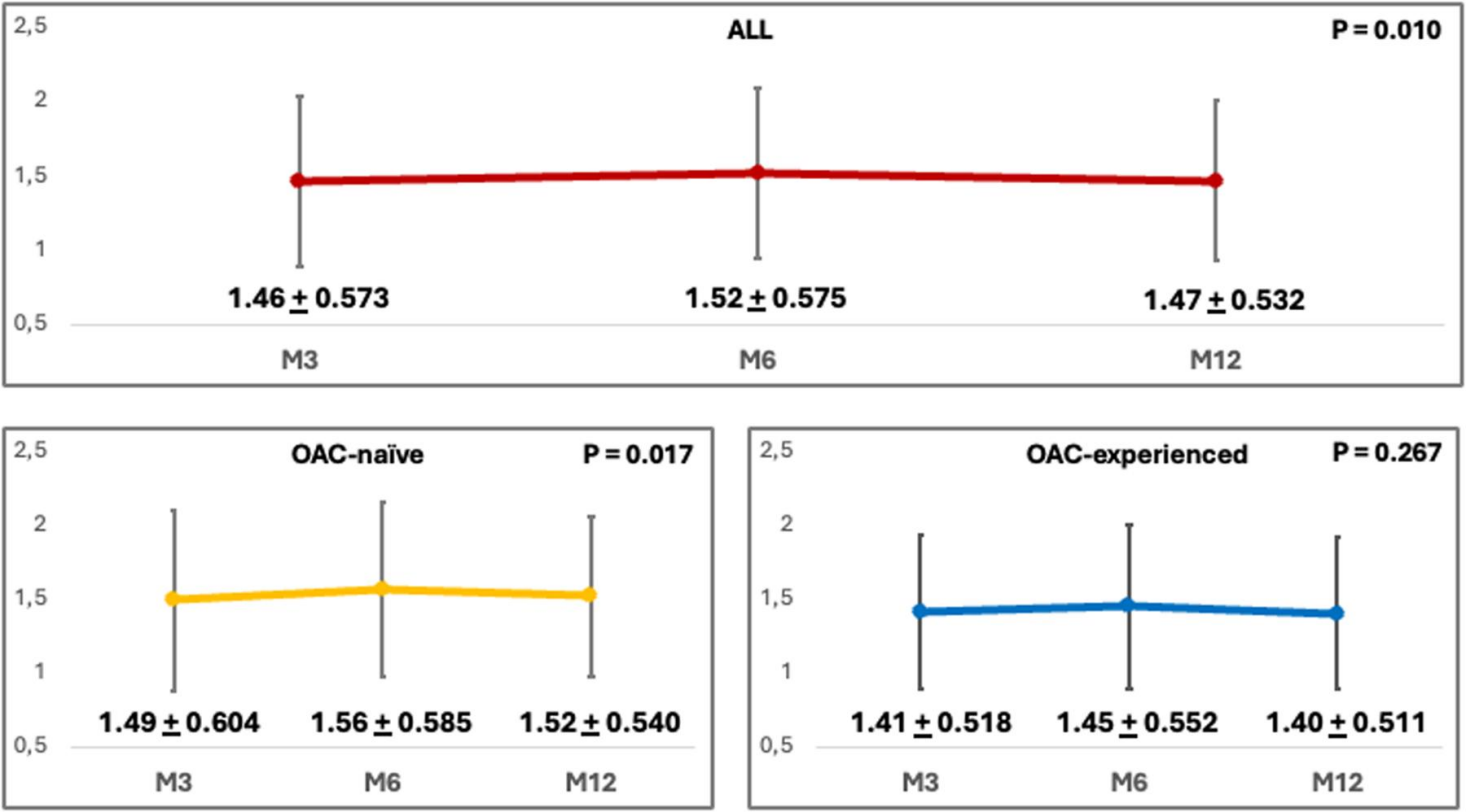
Time trends in patient self‐reported satisfaction with DOAC (mean value and standard deviation) during follow‐up: The whole study cohort (upper panel), OAC‐naïve patients (lower left panel) and OAC‐experienced patients (lower right panel). M, month; OAC, oral anticoagulant therapy. *The lower the mean value, the higher the level of satisfaction.

During follow‐up, statistically significant changes in the mean value of patient self‐reported global satisfaction with generic rivaroxaban were observed in the whole study cohort (Figure 2, upper panel) and among OAC‐naïve patients (Figure 2, lower left panel) but not within the OAC‐experienced group (Figure 2, lower right panel).

The pattern of change in the mean value of patient self‐reported global satisfaction with generic rivaroxaban was comparable between the whole study cohort and OAC‐naïve group (a decrease in the level of satisfaction at the 6‐month follow‐up visit, followed by an increase at 12 months), whereas the mean value of patient satisfaction in the OAC‐experienced group remained broadly constant (Figure 2).

**FIGURE 3:**
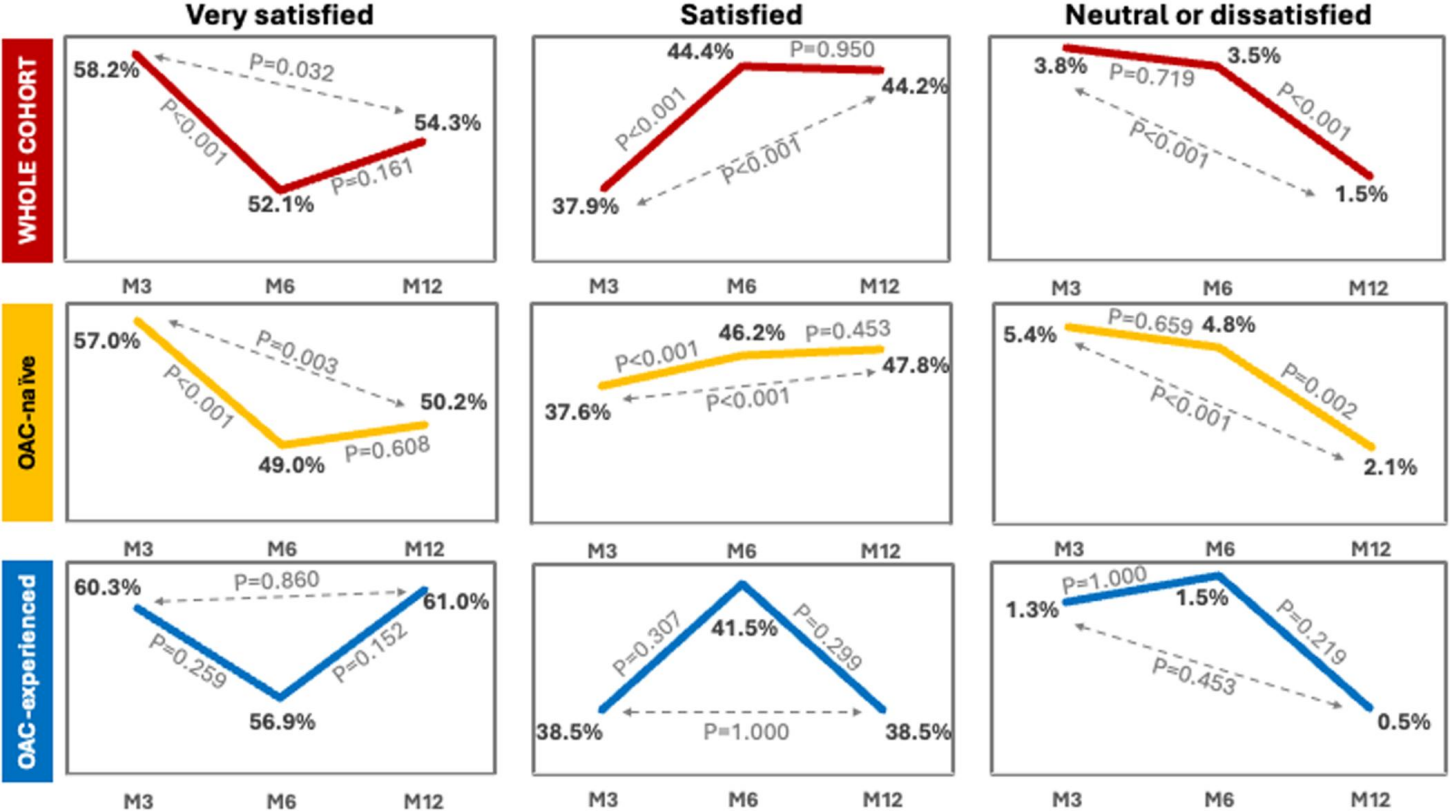
Changes in patient self‐reported satisfaction with DOAC per level of satisfaction and follow‐up visit. M, month; OAC, oral anticoagulant therapy.

A detailed insight into the changes in patient self‐reported global satisfaction per each level of satisfaction and follow‐up visit is shown in Figure 3.

In the OAC‐naïve group, the proportion of very satisfied patients statistically significantly declined at 6 months compared with the 3‐month follow‐up visit and then slightly (but not statistically significantly) increased at 12 months. The proportion of neutral/dissatisfied OAC‐naïve patients has declined at each follow‐up visit, with statistically significant decline at 12 months compared to both 6‐ and 3‐month follow‐up visit, while the proportion of satisfied OAC‐naïve patients has significantly increased at both 6‐ and 12‐month visit (Figure 3).

A pattern of changes in the OAC‐experienced group was broadly similar to that in the OAC‐naïve group, but all changes were statistically non‐significant. The changes in patient self‐reported global satisfaction with DOAC in the whole study group mostly reflected the changes observed among OAC‐naïve patients (Figure 3).

### 3.4 Sensitivity Analyses

Generic rivaroxaban was permanently discontinued in 21/1092 patients (1.9%) by the 3‐month follow‐up visit, in an additional 33 patients (3.0%) by the 6‐month follow‐up visit and in an additional 18 patients (1.7%) by the 12‐month follow‐up visit (a total of 72 patients, 6.6%). Accordingly, data on the global patient satisfaction with generic rivaroxaban therapy was available for 1071, 1038, 1020 patients at the 3‐, 6‐, and 12‐month follow‐up visits.

A comparison of demographic data and study cohort characteristics at enrollment between patients included in the main analysis (Group 1, *n* = 1020) and excluded patients (Group 2, *n* = 72) revealed no statistically significant differences, except a significantly greater proportion of patients with first‐diagnosed AF in Group 1 (*n* = 430, 42.2%) versus Group 2 (*n* = 20, 27.8%), *p* = 0.017 (see Supporting Information S1: Table 1).

Patients in Group 2 were statistically significantly less satisfied with generic rivaroxaban treatment than patients in Group 1 at both 3‐ and 6‐month follow‐up visits (1.84 ± 0.95 vs. 1.46 ± 0.57, *p* < 0.001 and 1.83 ± 0.79 vs. 1.52 + 0.58, *p* = 0.022, respectively) when comparing mean values. The observed difference in patient satisfaction was mostly driven by a significantly lower proportion of very satisfied patients, as well as a higher proportion of neutral or dissatisfied patients in Group 2 at the 3‐month follow‐up visit, whereas there was no statistically significant difference in the proportion of patients in different satisfaction categories at the 6‐month follow‐up visit (see Supporting Information S1: Table 2).

A sensitivity analysis of patient self‐reported satisfaction with DOAC therapy at the 3‐, 6‐, and 12‐month follow‐up visits comparing OAC‐naïve versus OAC‐experienced patients, which included the whole cohort of 1092 patients with all available follow‐up visit data per patient, revealed an identical pattern of differences as the one shown in Figure 1. (see Supporting Information S1: Table 3).

## 4 Discussion

The present study is the first study specifically comparing patient self‐reported global satisfaction with a DOAC therapy (i.e., generic rivaroxaban) in patients with and without prior experience with OAC in a relatively large cohort of patients with AF, over a 12‐month follow‐up.

The study main findings were as follows: (i) overall patient self‐reported global satisfaction with rivaroxaban was high across all follow‐up visits, (ii) on exploratory analyses, OAC‐naïve status was statistically significantly associated with the level of patient satisfaction with rivaroxaban during follow‐up, (iii) the mean level of global satisfaction with rivaroxaban was consistently statistically significantly lower in OAC‐naïve compared with OAC‐experienced patients at each follow‐up visit and (iv) statistically significant fluctuations in the levels of patient satisfaction with rivaroxaban during follow‐up were observed among OAC‐naïve patients but not in the OAC‐experienced group, thus suggesting that OAC‐naïve patients with AF need additional attention and more intense clinical follow‐up in the first year of OAC therapy initiation.

Similarly to previously published studies [19, 21], a high level of patient self‐reported satisfaction with DOAC therapy (i.e., generic rivaroxaban) was observed in the present study, with > 50% of participants being very satisfied with the therapy at each follow‐up visit. Nevertheless, our findings underscore a significant room for further improvement in patient satisfaction among AF patients at increased risk of stroke (the mean CHA_2_DS_2_‐VASc score value was 3.16 in our study), given that previously published studies suggested that patient satisfaction with therapy may substantially influence treatment adherence [12, 26] and, consequently, the risk of adverse health outcomes [5, 8].

A recent meta‐analysis of four randomized clinical trials and 16 observational studies with a total of 18 648 patients with AF or venous thromboembolism reported more favorable patient satisfaction scores with DOACs in comparison to VKAs, notwithstanding a considerable methodological heterogeneity among the studies [19]. However, potential differences between OAC‐naïve and OAC‐experienced patient groups were not addressed in the included studies.

In our study, the mean level of patient satisfaction with rivaroxaban was statistically significantly lower in OAC‐naïve patients compared with the OAC‐experienced group at each follow‐up visit and, on exploratory analyses, OAC‐naïve status was consistently significantly associated with specific levels of patient satisfaction across all follow‐up visits. At 3 months, OAC‐naïve patients had 3.9‐fold greater odds for being neutral to (or dissatisfied with) rivaroxaban therapy, using univariate analysis. At the 6‐month follow‐up visit, OAC‐naïve status was associated with 26% lower odds of being very satisfied, and at 12 months OAC‐naïve patients had 33% lower odds of being very satisfied but 39% higher odds of being satisfied with rivaroxaban on multivariate analyses, while the association with being neutral (or dissatisfied) was no longer statistically significant at either of those two follow‐up visits. Indeed, the proportion of patients being neutral (or dissatisfied) gradually decreased during the first year of treatment in OAC‐naïve patients, while the total proportion of patients being very satisfied or satisfied with rivaroxaban treatment gradually increased (from 94.6% at 3 months, to 95.2% at 6 months and 98.0% at 12 months). Our finding is in line with two recent studies, from Canada [21] and Japan [11], which consistently reported gradually increasing patient global satisfaction with OAC therapy over time although using different methodologies (again, data on prior experience with OAC therapy were not reported). In our study, minor fluctuations in the level of global patient satisfaction of comparable direction were also observed in the OAC‐experienced group, but those changes were not statistically significant.

During the initiation of OAC therapy, OAC‐naïve patients often exhibit higher rates of thromboembolic and bleeding events [22, 27] and greater risk of suboptimal adherence or non‐adherence to OAC therapy compared with OAC‐experienced patients [5, 9]. Notably, treatment with DOACs has been shown to be less tolerant to suboptimal adherence (and persistence) compared with VKAs [5, 28, 29]. In the present study, we observed consistently lower levels of patient global satisfaction with rivaroxaban among OAC‐naïve compared with the OAC‐experienced patients, thus possibly identifying a plausible reason for the need for additional support to OAC‐naïve patients as one of the key targets for interventions aiming to improve adherence to OAC therapy.

Similar to several previously published reports, a comparison of patient satisfaction with rivaroxaban between patients previously taking a VKA and those previously taking dabigatran or apixaban yielded no significant differences between these two subgroups [20].

Time trend analyses in the present study revealed significant changes in the levels of patient global satisfaction with rivaroxaban across follow‐up visits in OAC‐naïve participants, especially at 6 months since the DOAC initiation, with a significant decline in the proportion of very satisfied patients and only a slight decrease in the proportion of neutral (or dissatisfied) patients as compared to the 3‐month follow‐up visit (from 57.0% to 49.0%, *p* < 0.001 and from 5.4% to 4.8%, *p* = 0.659, respectively). These findings suggest that OAC‐naïve patients need additional support and intense clinical follow‐up especially in the first 6 months of treatment with a DOAC.

Fluctuations in the level of patient satisfaction with DOAC therapy in OAC‐naïve patients could be, at least partly, attributed to a lack of previous familiarity with the concept of a long‐term treatment to prevent adverse clinical outcomes. Indeed, 66% of OAC‐naïve patients in our study had a first‐diagnosed AF and might not have previously experienced any long‐term therapy for a medical condition. In addition, patients in our study were neutral, rather than dissatisfied or very dissatisfied, which also reflects a lack of adequate reference in the patient's previous experience with various therapies.

In addition to OAC‐naïve status, hyperlipidaemia and renal impairment were most consistently associated with specific levels of patient satisfaction with rivaroxaban on multivariate analyses in our study. Possibly, patients with hyperlipidaemia and/or renal impairment could be more familiar with the concept of long‐term therapy for the prevention of major adverse outcomes compared with other patients. The previously mentioned Canadian study [21] also explored factors associated with AF patent satisfaction with OAC therapy. In that study, treatment with a VKA, female sex, low stroke risk and no bleeding events during follow‐up were significantly associated with greater global patient satisfaction with OAC, whereas data on previous OAC status (i.e., OAC‐naïve or experienced) was not reported.

### 4.1 Clinical Implications

Our study corroborates increasing perception of clinical importance of considering patient‐reported outcome measures [20, 30].

Main findings of the present study could have some clinically relevant implications in the modern era of personalized, patient‐centered management of AF. A high level of patient self‐reported satisfaction with DOAC therapy may abolish (or diminish) physicians' reluctance to prescribe an OAC in some AF patients, especially if there is a concern about the patient's adherence to therapy. Selecting the OAC agent shown to be associated with greater levels of patient satisfaction could also improve the patient's adherence to OAC therapy and, consequently, reduce the risk of adverse clinical outcomes. Finally, whichever OAC agent is prescribed, patients with AF require substantial support in terms of shared informed treatment decision making, regular periodic clinical follow‐up visits, reassurance, clarification of misconceptions regarding OAC‐related risks and benefits, and so on [31, 32, 33]. Our results suggest that OAC‐naïve patients would require additional support and more intense regular clinical follow‐up within the first year of OAC initiation, especially in the first 6 months of OAC therapy. In addition, OAC‐naïve patients can be reassured that their global satisfaction with recently initiated DOAC therapy would generally improve over time.

### 4.2 Study Limitations

The present study is limited by its observational design, and potential selection bias could be further introduced by selecting only the patients who completed the 12‐month follow‐up. However, such patient selection was conditioned by the aim of the present study to explore potential differences in patient self‐reported global satisfaction with rivaroxaban at pre‐defined follow‐up time points and compare time trends in patient satisfaction with the DOAC therapy between OAC‐naïve and OAC‐experienced patients with AF. Notably, the participants included in the present study represent 93.4% of the original Guardian study population. In addition, we have compared demographics and study cohort characteristics at enrollment between the patients included in the main analysis and excluded patients and conducted sensitivity analyses including the whole Guardian study cohort.

Notably, the prevalence of first‐diagnosed AF was significantly higher among patients included in the main analysis, as well as among OAC‐naïve patients (compared to OAC‐experienced) thus suggesting potentially strong association between the OAC‐naïve status and first‐diagnosed AF. Whereas collinearity testing showed a significant relationship between first‐diagnosed AF and OAC‐naïve status, there was an acceptable level of independence of the two variables.

In addition, OAC‐experienced patients were significantly older, more often with comorbidity or multimorbidity and at higher stroke and bleeding risk compared to OAC‐naïve patients. Despite adjustments for the observed differences, there is a great potential for residual confounding. Moreover, potential center‐ or country‐specific effects could not be excluded.

Since rivaroxaban was prescribed to all participants at enrollment, we analyzed only a modified TSQM‐II domain addressing patient global satisfaction, while other TSQM‐II domains (i.e., patient satisfaction with effectiveness, convenience and adverse events) were deemed less relevant in the absence of comparator OAC drug(s). The use of a modified patient self‐reported satisfaction with treatment assessment tool is also a limitation, since it precludes direct comparisons with other studies and may not completely capture relevant information. Finally, adherence to therapy was not assessed in the present study, and we have not explored the association of adverse clinical outcomes with patient satisfaction, owing to the very low rates of adverse clinical outcomes observed in the Guardian study cohort [24].

## 5 Conclusions

Our study showed persistently high levels of overall patient self‐reported global satisfaction with rivaroxaban over 12 months. Compared with OAC‐experienced patients with AF, OAC‐naïve patients consistently had significantly lower levels of patient satisfaction with the DOAC across all follow‐up visits, with significant fluctuations in the level of satisfaction especially at 6 months after treatment initiation and an increase in the level of patient satisfaction thereafter. Our results suggest that OAC‐naïve patients need additional support and more intense regular clinical follow‐up, particularly in the first 6 months after initiation of OAC therapy. However, OAC‐naive patients can be reassured that their satisfaction with recently initiated (D)OAC therapy would generally improve with time. Further research is needed to better define the factors associated with and clinical consequences of fluctuations of patient self‐reported satisfaction with (D)OAC therapy in OAC‐naïve patients.

## Author Contributions

Conceptualization: BM and TP; Methodology: BM and TP; Formal Analysis: BM; Investigation: BM, MM, AK, JS, MilanM, VK, MI, NI, NM, BP, TP; Writing – Original Draft Preparation: BM; Writing – Review and editing: BM, MM, AK, JS, MilanM, VK, MI, NI, NM, BP, TP; Visualization: BM, TP; Supervision: TP; Funding Acquisition: NM, TP, BP.

## Consent

At enrollment, all participants provided a written informed consent. Patients with AF prescribed OAC for the prevention of AF‐related thromboembolic events, who were never taking OAC before for any indication. The study was approved by respective Institutional Review Board in each of 14 participating centers.

## Conflicts of Interest

The authors declare no conflicts of interest.

## Supporting information


Supporting File

## Data Availability

The data that support the findings of this study are available on request from the corresponding author. The data are not publicly available due to privacy or ethical restrictions.
