# Supplementary material for: Patient Self‐Reported Global Satisfaction With Rivaroxaban in Patients With Atrial Fibrillation: A Comparison Between Oral Anticoagulation‐Naïve Versus Oral Anticoagulation‐Experienced Patients
Source: Clin Cardiol. 2026 Sep 2;49(9):e70461. doi: 10.1002/clc.70461 (PMC13536027; doi:10.1002/clc.70461)
Supplement: Supplementary file 1 [file CLC-49-e70461-s001.docx]

**Patient self-reported global satisfaction with rivaroxaban in patients with atrial fibrillation: A comparison between oral anticoagulation-naïve versus oral anticoagulation-experienced patients**

**Supplemental material**

Bogdan Markovic^1^, Miroslav Mihajlovic^2^, Aleksandar Kocijancic^1,2^, Jelena Simic^1,2^, Milan Marinkovic^1,2^, Vladan Kovacevic^1,2^, Milica Ivanisevic^2^, Nikola Isailovic^2^, Nebojsa Mujovic^1,2^, Belma Pojskic^3^, Tatjana Potpara^1,2^, on behalf of the GUARDIAN Investigators.

^1^ Medical Faculty, University of Belgrade, Belgrade, Serbia.

^2^ Cardiology Clinic, University Clinical Centre of Serbia, Belgrade, Serbia.

^3^ Department of General Internal medicine, Zenica Cantonal Hospital; Faculty of medicine, University of Zenica, Zenica, Bosnia and Herzegovina.

**Corresponding Author:**

Professor Tatjana Potpara, MD, PhD, FESC

Medical Faculty, University of Belgrade, Dr Subotica starijeg 8, 11000 Belgrade, Serbia.

Tel: +381113616319

Email: tatjana.potpara@med.bg.ac.rs

**Table S1. Demographic data and study cohort characteristics at enrolment (overall and comparison between patients included in the main analyses (Group1) and patients excluded from the main analyses (Group 2)).**

| **Variable** | **All patients**  **N=1092 (100%)** | **Group 1**  **N=1020 (93.4%)** | **Group 2**  **N=72  (6.6%)** | **P value** |
| --- | --- | --- | --- | --- |
| **Age (years)** | 67.93 ± 9.75 (range 31-90) | 68.02 ± 9.65 (range 31-90) | 66.79 ± 11.01 (range 31-90) | 0.303 |
| **Age under 65 years** | 333 (30.5) | 308 (30.2) | 25 (34.7) | 0.420 |
| **Age 65-74 years** | 488 (44.7) | 460 (45.1) | 28 (38.9) | 0.306 |
| **Age 75-80 years** | 183 (16.8) | 173 (17.0) | 10 (13.9) | 0.500 |
| **Age over 80 years** | 88 (8.1%) | 79 (7.7) | 9 (12.5) | 0.152 |
| **Female sex** | 468 (42.9) | 445 (43.6) | 49 (68.1) | 0.053 |
| **First-diagnosed AF** | 450 (41.2) | 430 (42.2) | 20 (27.8) | 0.017 |
| **CHA_2_DS_2_-VASc score** | 3.15 ± 1.55 (range 0-9) | 3.16 ± 1.54 (range 0-9) | 3.04 ± 1.68 (range 0-9) | 0.533 |
| **HAS-BLED score** | 1.18 ± 0.92 (range 0-6) | 1.19 ± 0.92 (range 0-6) | 0.97 ± 0.86 (range 0-6) | 0.051 |
| **Body Mass Index** | 27.68 ± 3.78 (range 18-50) | 27.66 ± 3.74 (range 20-50) | 27.94 ± 4.36 (range 18-50) | 0.551 |
| **Overweight or obese** | 842 (77.1) | 785 (77.0) | 57 (79.2) | 0.667 |
| **Current smoker** | 191 (17.5) | 183 (17.9) | 8 (11.1) | 0.140 |
| **Hyperlipidaemia** | 531 (48.6) | 502 (49.2) | 29 (40.3) | 0.143 |
| **Any comorbidity** | 1045 (95.7) | 976 (95.7) | 69 (95.8) | 0.143 |
| **Hypertension** | 963 (88.2) | 903 (88.5) | 60 (83.3%) | 0.187 |
| **Coronary artery disease** | 254 (23.3) | 232 (22.7) | 22 (30.6) | 0.130 |
| **Diabetes mellitus** | 250 (22.9) | 232 (22.7) | 18 (25.0) | 0.660 |
| **Heart failure** | 281 (25.7) | 262 (25.7) | 19 (26.4) | 0.895 |
| **Prior stroke / TIA** | 94 (8.6) | 89 (8.7) | 5 (6.9) | 0.603 |
| **Renal impairment** | 79 (7.2) | 38 (6.0) | 2 (2.8) | 0.131 |
| **Obstructive sleep apnoea** | 7 (0.6) | 6 (0.6) | 1 (1.4) | 0.380 |
| **Multimorbidity** | 787 (72.1) | 434 (68.9) | 51 (70.8) | 0.809 |

AF- Atrial Fibrillation; CHA_2_DS_2_-VASc score, Congestive Heart Failure History, Hypertension History, Age ≥ 75 Years, Age 65 to 74 Years, Diabetes Mellitus history, Stroke History, Vascular Disease History, female sex; HAS-BLED, Hypertension, Abnormal Renal Function, Abnormal Liver Function, Stroke History, Bleeding History or Predisposition, Labile INR, Elderly > 65 Years, Drugs or Alcohol; BMI, Body Mass Index, TIA, Transient Ischemic Attack; OAC- oral anticoagulant therapy;

**Table S2. Patient self-reported satisfaction with DOAC therapy at the 3-, 6- and 12-month follow-up visit: comparison of patients included in the main analyses (Group1) and patients excluded from the main analyses (Group 2)**

| **Variable** | **All patients**  **N=1071^a^ (100%)** | **Group 1**  **N=1020  (95.2 %)** | **Group 2**  **N=51 (4.8%)** | **P value** |
| --- | --- | --- | --- | --- |
| **M3: Very Satisfied** | 615 (57.4) | 594 (58.2) | 21 (41.2) | 0.016 |
| **M3: Satisfied** | 409 (38.2) | 387 (37.9) | 22 (43.1) | 0.456 |
| **M3: Neutral or dissatisfied** | 47 (4.4) | 39 (3.8) | 8 (15.7) | 0.001 |
|  | **All patients**  **N=1038^b^ (100%)** | **Group 1**  **N=1020 (98.3%)** | **Group 2**  **N=18 (1.7)** | **P value** |
| **M6: Very satisfied** | 537 (51.7) | 531 (52.1) | 6 (33.3) | 0.115 |
| **M6: Satisfied** | 463 (44.6) | 453 (44.4) | 10 (55.6) | 0.346 |
| **M6: Neutral or dissatisfied** | 38 (3.7) | 36 (3.5) | 2 (11.1) | 0.138 |

^a^ 21 patients permanently discontinued rivaroxaban by the 3-month follow-up visit.

^b^ A total of 54 patients permanently discontinued rivaroxaban by the 6-month follow-up visit.

M, month.

**Table S3. Patient self-reported satisfaction with DOAC therapy at the 3-, 6- and 12-month follow-up visit: comparison of the OAC-naïve versus OAC-experienced group in the whole study cohort (Group 1 plus Group 2)**

| **Variable** | **All patients**  **N=1071^a^ (100%)** | **OAC-naïve**  **N= 656 (61.3%)** | **OAC-experienced**  **N=415 (38.7%)** | **P value** |
| --- | --- | --- | --- | --- |
| **M3: Very Satisfied** | 615 (57.4) | 365 (55.6) | 250 (60.2) | 0.138 |
| **M3: Satisfied** | 409 (38.2) | 252 (38.4) | 157 (37.8) | 0.848 |
| **M3: Neutral or dissatisfied** | 47 (4.4) | 39 (5.9) | 8 (1.9) | 0.002 |
|  | **All patients**  **N=1038^b^ (100%)** | **OAC-naïve**  **N=641 (61.8%)** | **OAC-experienced**  **N=397 (38.2%)** | **P value** |
| **M6: Very satisfied** | 537 (51.7) | 312 (48.7) | 225 (56.7) | 0.012 |
| **M6: Satisfied** | 463 (44.6) | 299 (46.6) | 164 (41.3) | 0.093 |
| **M6: Neutral or dissatisfied** | 38 (3.7) | 30 (4.7) | 8 (2.0) | 0.026 |
|  | **All patients**  **N=1020^c^ (100%)** | **OAC-naïve**  **N=630 (61.8%)** | **OAC-experienced**  **N=390 (38.2%)** | **P value** |
| **M12: Very satisfied** | 554 (54.3) | 316 (50.2) | 238 (61.0) | 0.001 |
| **M12: Satisfied** | 451 (44.2) | 301 (47.8) | 150 (38.5) | 0.004 |
| **M12: Neutral or dissatisfied** | 15 (1.5) | 13 (2.1) | 2 (0.5) | 0.046 |

^a^ 21 patients permanently discontinued rivaroxaban by the 3-month follow-up visit.

^b^ A total of 54 patients permanently discontinued rivaroxaban by the 6-month follow-up visit.

^c^ A total of 72 patients permanently discontinued rivaroxaban by the 12-month follow-up visit.

M, month.

**Table S4. Univariate binary logistic regression of patient satisfaction at Month 3: Very satisfied**

| **Variable** | **Odds ratio** | **95% CI** | **P value** |
| --- | --- | --- | --- |
| Age | 0.994 | 0.981-1.007 | 0.339 |
| Age under 65 | 1.114 | 0.849-1.463 | 0.436 |
| Age 65-74 | 1.018 | 0.793-1.308 | 0.887 |
| Age 75-80 | 1.098 | 0.787-1.533 | 0.582 |
| Age over 80 | 0.575 | 0.362-0.912 | **0.019** |
| Female sex | 0.780 | 0.607-1.003 | 0.053 |
| First-diagnosed AF | 0.856 | 0.666-1.101 | 0.226 |
| CHA_2_DS_2_-VASc score | 0.965 | 0.890-1.046 | 0.389 |
| HAS-BLED score | 1.029 | 0.899-1.179 | 0.678 |
| BMI | 1.030 | 0.995-1.065 | 0.094 |
| Overweight or obese | 1.091 | 0.813-1.465 | 0.561 |
| Current smoker | 0.907 | 0.657-1.253 | 0.555 |
| Hyperlipidaemia | 1.728 | 1.343-2.222 | **<0.001** |
| Any comorbidity | 0.639 | 0.335-1.220 | 0.175 |
| Hypertension | 1.272 | 0.864-1.872 | 0.222 |
| Coronary artery disease | 1.094 | 0.812-1.474 | 0.555 |
| Diabetes mellitus | 1.120 | 0.831-1.509 | 0.459 |
| Heart failure | 0.927 | 0.698-1.232 | 0.603 |
| Prior stroke / TIA | 1.211 | 0.783-1.875 | 0.390 |
| Renal impairment | 0.953 | 0.596-1.524 | 0.840 |
| Obstructive sleep apnoea | 3.608 | 0.420-30.993 | 0.242 |
| Multimorbidity | 1.332 | 1.011-1.755 | **0.042** |
| OAC-naive | 0.874 | 0.676-1.130 | 0.303 |
| OAC-experienced | 1.144 | 0.885-1.480 | 0.303 |

AF- Atrial Fibrillation; CHA_2_DS_2_-VASc score, Congestive Heart Failure History, Hypertension History, Age ≥ 75 Years, Age 65 to 74 Years, Diabetes Mellitus history, Stroke History, Vascular Disease History, female sex; HAS-BLED, Hypertension, Abnormal Renal Function, Abnormal Liver Function, Stroke History, Bleeding History or Predisposition, Labile INR, Elderly > 65 Years, Drugs or Alcohol; BMI, Body Mass Index, TIA, Transient Ischemic Attack; OAC- oral anticoagulant therapy;

**Table S5. Univariate binary logistic regression of patient satisfaction at Month 3: Satisfied**

| **Variable** | **Odds ratio** | **95% CI** | **P value** |
| --- | --- | --- | --- |
| Age | 1.009 | 0.996-1.023 | 0.163 |
| Age under 65 | 0.908 | 0.688-1.198 | 0.495 |
| Age 65-74 | 0.896 | 0.694-1.156 | 0.397 |
| Age 75-80 | 1.041 | 0.744-1.457 | 0.815 |
| Age over 80 | 1.756 | 1.108-2.783 | **0.017** |
| Female sex | 1.228 | 0.952-1.584 | 0.114 |
| First-diagnosed AF | 1.015 | 0.785-1.311 | 0.911 |
| CHA_2_DS_2_-VASc score | 1.052 | 0.970-1.142 | 0.223 |
| HAS-BLED score | 1.017 | 0.886-1.166 | 0.812 |
| BMI | 0.970 | 0.936-1.004 | 0.082 |
| Overweight or obese | 0.958 | 0.710-1.292 | 0.778 |
| Current smoker | 1.105 | 0.797-1.533 | 0.549 |
| Hyperlipidaemia | 0.675 | 0.523-0.871 | **0.003** |
| Any comorbidity | 1.664 | 0.846-3.271 | 0.140 |
| Hypertension | 0.767 | 0.519-1.132 | 0.182 |
| Coronary artery disease | 0.887 | 0.654-1.202 | 0.439 |
| Diabetes mellitus | 0.999 | 0.739-1.351 | 0.997 |
| Heart failure | 1.081 | 0.810-1.442 | 0.596 |
| Prior stroke / TIA | 0.805 | 0.518-1.250 | 0.334 |
| Renal impairment | 1.111 | 0.692-1.785 | 0.663 |
| Obstructive sleep apnoea | 0.325 | 0.038-2.796 | 0.306 |
| Multimorbidity | 0.794 | 0.600-1.050 | 0.106 |
| OAC-naive | 0.965 | 0.744-1.251 | 0.788 |
| OAC-experienced | 1.036 | 0.799-1.344 | 0.788 |

AF- Atrial Fibrillation; CHA_2_DS_2_-VASc score, Congestive Heart Failure History, Hypertension History, Age ≥ 75 Years, Age 65 to 74 Years, Diabetes Mellitus history, Stroke History, Vascular Disease History, female sex; HAS-BLED, Hypertension, Abnormal Renal Function, Abnormal Liver Function, Stroke History, Bleeding History or Predisposition, Labile INR, Elderly > 65 Years, Drugs or Alcohol; BMI, Body Mass Index, TIA, Transient Ischemic Attack; OAC- oral anticoagulant therapy;

**Table S6. Univariate binary logistic regression of patient satisfaction at Month 3: Neutral, dissatisfied or very dissatisfied**

| **Variable** | **Odds ratio** | **95% CI** | **P value** |
| --- | --- | --- | --- |
| Age | 0.983 | 0.953-1.014 | 0.282 |
| Age under 65 | 0.905 | 0.445-1.841 | 0.783 |
| Age 65-74 | 1.789 | 0.934-3.429 | 0.079 |
| Age 75-80 | 0.256 | 0.061-1.073 | 0.062 |
| Age over 80 | 0.992 | 0.299-3.297 | 0.990 |
| Female sex | 1.377 | 0.726-2.613 | 0.327 |
| First-diagnosed AF | 2.540 | 1.304-4.946 | **0.006** |
| CHA_2_DS_2_-VASc score | 0.910 | 0.735-1.126 | 0.384 |
| HAS-BLED score | 0.713 | 0.480-1.060 | 0.094 |
| BMI | 1.004 | 0.922-1.093 | 0.925 |
| Overweight or obese | 0.753 | 0.369-1.537 | 0.436 |
| Current smoker | 1.001 | 0.435-2.304 | 0.999 |
| Hyperlipidaemia | 0.297 | 0.140-0.632 | **0.002** |
| Any comorbidity | 0.827 | 0.193-3.549 | 0.799 |
| Hypertension | 1.139 | 0.397-3.265 | 0.808 |
| Coronary artery disease | 1.179 | 0.566-2.457 | 0.660 |
| Diabetes mellitus | 0.377 | 0.133-1.073 | 0.068 |
| Heart failure | 0.998 | 0.479-2.076 | 0.995 |
| Prior stroke / TIA | 1.153 | 0.348-3.822 | 0.816 |
| Renal impairment | 0.653 | 0.154-2.762 | 0.562 |
| Obstructive sleep apnoea | 0.000 | 0.000-NR | 0.999 |
| Multimorbidity | 0.678 | 0.347-1.324 | 0.255 |
| OAC-naive | 4.393 | 1.703-11.329 | **0.002** |
| OAC-experienced | 0.228 | 0.088-0.587 | **0.002** |

AF- Atrial Fibrillation; CHA_2_DS_2_-VASc score, Congestive Heart Failure History, Hypertension History, Age ≥ 75 Years, Age 65 to 74 Years, Diabetes Mellitus history, Stroke History, Vascular Disease History, female sex; HAS-BLED, Hypertension, Abnormal Renal Function, Abnormal Liver Function, Stroke History, Bleeding History or Predisposition, Labile INR, Elderly > 65 Years, Drugs or Alcohol; BMI, Body Mass Index, TIA, Transient Ischemic Attack; OAC- oral anticoagulant therapy;

**Table S7. Univariate binary logistic regression of patient satisfaction at Month 6: Very satisfied**

| **Variable** | **Odds ratio** | **95% CI** | **P value** |
| --- | --- | --- | --- |
| Age | 0.988 | 0.975-1.000 | 0.057 |
| Age under 65 | 1.154 | 0.882-1.509 | 0.296 |
| Age 65-74 | 1.127 | 0.880-1.443 | 0.343 |
| Age 75-80 | 0.821 | 0.592-1.139 | 0.239 |
| Age over 80 | 0.638 | 0.401-1.016 | 0.058 |
| Female sex | 0.791 | 0.617-1.014 | 0.064 |
| First-diagnosed AF | 0.800 | 0.624-1.026 | 0.079 |
| CHA_2_DS_2_-VASc score | 0.952 | 0.879-1.031 | 0.226 |
| HAS-BLED score | 0.933 | 0.816-1.066 | 0.307 |
| BMI | 1.022 | 0.989-1.057 | 0.192 |
| Overweight or obese | 0.985 | 0.736-1.319 | 0.922 |
| Current smoker | 0.781 | 0.567-1.076 | 0.131 |
| Hyperlipidaemia | 1.407 | 1.099-1.800 | **0.007** |
| Any comorbidity | 0.991 | 0.541-1.814 | 0.977 |
| Hypertension | 0.820 | 0.557-1.209 | 0.317 |
| Coronary artery disease | 1.258 | 0.937-1.689 | 0.127 |
| Diabetes mellitus | 0.879 | 0.656-1.178 | 0.388 |
| Heart failure | 1.034 | 0.780-1.369 | 0.818 |
| Prior stroke / TIA | 0.968 | 0.626-1.496 | 0.882 |
| Renal impairment | 0.889 | 0.559-1.415 | 0.621 |
| Obstructive sleep apnoea | 4.639 | 0.540-39.846 | 0.162 |
| Multimorbidity | 1.189 | 0.904-1.563 | 0.216 |
| OAC-naive | 0.728 | 0.565-0.939 | **0.015** |
| OAC-experienced | 1.373 | 1.065-1.770 | **0.015** |

AF- Atrial Fibrillation; CHA_2_DS_2_-VASc score, Congestive Heart Failure History, Hypertension History, Age ≥ 75 Years, Age 65 to 74 Years, Diabetes Mellitus history, Stroke History, Vascular Disease History, female sex; HAS-BLED, Hypertension, Abnormal Renal Function, Abnormal Liver Function, Stroke History, Bleeding History or Predisposition, Labile INR, Elderly > 65 Years, Drugs or Alcohol; BMI, Body Mass Index, TIA, Transient Ischemic Attack; OAC- oral anticoagulant therapy;

**Table S8. Univariate binary logistic regression of patient satisfaction at Month 6: Satisfied**

| **Variable** | **Odds ratio** | **95% CI** | **P value** |
| --- | --- | --- | --- |
| Age | 1.019 | 1.005-1.032 | 0.006 |
| Age under 65 | 0.831 | 0.634-1.089 | 0.179 |
| Age 65-74 | 0.834 | 0.650-1.070 | 0.153 |
| Age 75-80 | 1.329 | 0.958-1.845 | 0.088 |
| Age over 80 | 1.829 | 1.148-2.913 | **0.011** |
| Female sex | 1.182 | 0.922-1.516 | 0.188 |
| First-diagnosed AF | 1.085 | 0.845-1.394 | 0.521 |
| CHA_2_DS_2_-VASc score | 1.058 | 0.976-1.146 | 0.170 |
| HAS-BLED score | 1.052 | 0.920-1.203 | 0.458 |
| BMI | 0.975 | 0.943-1.009 | 0.144 |
| Overweight or obese | 0.943 | 0.703-1.264 | 0.694 |
| Current smoker | 1.230 | 0.893-1.695 | 0.205 |
| Hyperlipidaemia | 0.751 | 0.587-0.963 | **0.024** |
| Any comorbidity | 1.054 | 0.573-1.939 | 0.867 |
| Hypertension | 1.124 | 0.761.1.659 | 0.558 |
| Coronary artery disease | 0.833 | 0.619-1.121 | 0.227 |
| Diabetes mellitus | 1.170 | 0.872-1.569 | 0.295 |
| Heart failure | 1.056 | 0.797-1.401 | 0.703 |
| Prior stroke / TIA | 1.135 | 0.730-1.764 | 0.573 |
| Renal impairment | 1.311 | 0.824-2.088 | 0.253 |
| Obstructive sleep apnoea | 0.249 | 0.029-2.136 | 0.205 |
| Multimorbidity | 0.891 | 0.677-1.173 | 0.409 |
| OAC-Naive | 1.208 | 0.936-1.559 | 0.146 |
| OAC-experienced | 0.828 | 0.641-1.068 | 0.146 |

AF- Atrial Fibrillation; CHA_2_DS_2_-VASc score, Congestive Heart Failure History, Hypertension History, Age ≥ 75 Years, Age 65 to 74 Years, Diabetes Mellitus history, Stroke History, Vascular Disease History, female sex; HAS-BLED, Hypertension, Abnormal Renal Function, Abnormal Liver Function, Stroke History, Bleeding History or Predisposition, Labile INR, Elderly > 65 Years, Drugs or Alcohol; BMI, Body Mass Index, TIA, Transient Ischemic Attack; OAC- oral anticoagulant therapy;

**Table S9. Univariate binary logistic regression of patient satisfaction at Month 6: Neutral, dissatisfied or very dissatisfied**

| **Variable** | **Odds ratio** | **95% CI** | **P value** |
| --- | --- | --- | --- |
| Age | 0.964 | 0.935-0.994 | 0.021 |
| Age under 65 | 1.320 | 0.660-2.641 | 0.433 |
| Age 65-74 | 1.545 | 0.791-3.018 | 0.202 |
| Age 75-80 | 0.435 | 0.132-1.436 | 0.172 |
| Age over 80 | 0.001 | 0.001-NR | 0.997 |
| Female sex | 1.644 | 0.842-3.211 | 0.145 |
| First-diagnosed AF | 2.847 | 1.408-5.759 | 0.004 |
| CHA_2_DS_2_-VASc score | 0.955 | 0.768-1.188 | 0.680 |
| HAS-BLED score | 1.143 | 0.812-1.609 | 0.444 |
| BMI | 1.018 | 0.935-1.108 | 0.684 |
| Overweight or obese | 1.891 | 0.727-4.920 | 0.191 |
| Current smoker | 1.321 | 0.592-2.947 | 0.497 |
| Hyperlipidaemia | 0.647 | 0.327-1.279 | 0.210 |
| Any comorbidity | 0.758 | 0.176-3.261 | 0.710 |
| Hypertension | 2.250 | 0.533-9.489 | 0.270 |
| Coronary artery disease | 0.671 | 0.276-1.632 | 0.379 |
| Diabetes mellitus | 0.814 | 0.352-1.884 | 0.631 |
| Heart failure | 0.456 | 0.176-1.186 | 0.107 |
| Prior stroke / TIA | 0.579 | 0.219-1.528 | 0.269 |
| Renal impairment | 0.001 | 0.001-??? | 0.997 |
| Obstructive sleep apnoea | 0.001 | 0.001-NR | 0.999 |
| Multimorbidity | 0.672 | 0.336-1.346 | 0.263 |
| OAC-naive | 3.200 | 1.320-7.760 | 0.010 |
| OAC-experienced | 0.313 | 0.129-0.758 | 0.010 |

AF- Atrial Fibrillation; CHA_2_DS_2_-VASc score, Congestive Heart Failure History, Hypertension History, Age ≥ 75 Years, Age 65 to 74 Years, Diabetes Mellitus history, Stroke History, Vascular Disease History, female sex; HAS-BLED, Hypertension, Abnormal Renal Function, Abnormal Liver Function, Stroke History, Bleeding History or Predisposition, Labile INR, Elderly > 65 Years, Drugs or Alcohol; BMI, Body Mass Index, TIA, Transient Ischemic Attack; OAC- oral anticoagulant therapy;

**Table S10. Univariate binary logistic regression of patient satisfaction at Month 12: Very satisfied**

| **Variable** | **Odds ratio** | **95% CI** | **P value** |
| --- | --- | --- | --- |
| Age | 0.993 | 0.980-1.005 | 0.258 |
| Age under 65 | 1.156 | 0.883-1.513 | 0.291 |
| Age 65-74 | 1.069 | 0.834-1.369 | 0.600 |
| Age 75-80 | 0.778 | 0.561-1.080 | 0.134 |
| Age over 80 | 0.852 | 0.538-1.349 | 0.494 |
| Female sex | 0.790 | 0.616-1.013 | 0.063 |
| First-diagnosed AF | 0.728 | 0.567-0.935 | 0.013 |
| CHA_2_DS_2_-VASc score | 0.962 | 0.888-1.042 | 0.338 |
| HAS-BLED score | 1.076 | 0.941-1.231 | 0.286 |
| BMI | 1.036 | 1.002-1.071 | 0.040 |
| Overweight or obese | 1.185 | 0.885-1.587 | 0.255 |
| Current smoker | 0.699 | 0.507-0.963 | 0.029 |
| Hyperlipidaemia | 1.569 | 1.224-2.010 | <0.001 |
| Any comorbidity | 0.816 | 0.442-1.508 | 0.516 |
| Hypertension | 0.776 | 0.524-1.148 | 0.204 |
| Coronary artery disease | 1.094 | 0.815-1.469 | 0.549 |
| Diabetes mellitus | 1.023 | 0.762-1.372 | 0.882 |
| Heart failure | 1.080 | 0.814-1.432 | 0.595 |
| Prior stroke / TIA | 0.968 | 0.625-1.498 | 0.883 |
| Renal impairment | 1.827 | 1.115-2.994 | 0.017 |
| Obstructive sleep apnoea | 4.235 | 0.493-36.378 | 0.188 |
| Multimorbidity | 1.297 | 0.986-1.706 | 0.063 |
| OAC-naive | 0.643 | 0.497-0.831 | 0.001 |
| OAC-experienced | 1.556 | 1.204-2.011 | 0.001 |

AF- Atrial Fibrillation; CHA_2_DS_2_-VASc score, Congestive Heart Failure History, Hypertension History, Age ≥ 75 Years, Age 65 to 74 Years, Diabetes Mellitus history, Stroke History, Vascular Disease History, female sex; HAS-BLED, Hypertension, Abnormal Renal Function, Abnormal Liver Function, Stroke History, Bleeding History or Predisposition, Labile INR, Elderly > 65 Years, Drugs or Alcohol; BMI, Body Mass Index, TIA, Transient Ischemic Attack; OAC- oral anticoagulant therapy;

**Table S11. Univariate binary logistic regression of patient satisfaction at Month 12: Satisfied**

| **Variable** | **Odds ratio** | **95% CI** | **P value** |
| --- | --- | --- | --- |
| Age | 1.008 | 0.996-1.022 | 0.198 |
| Age under 65 | 0.840 | 0.641-1.101 | 0.207 |
| Age 65-74 | 0.947 | 0.739-1.214 | 0.667 |
| Age 75-80 | 1.269 | 0.915-1.761 | 0.154 |
| Age over 80 | 1.252 | 0.791-1.982 | 0.338 |
| Female sex | 1.238 | 0.965-1.589 | 0.093 |
| First-diagnosed AF | 1.295 | 1.008-1.663 | 0.043 |
| CHA_2_DS_2_-VASc score | 1.036 | 0.956-1.122 | 0.393 |
| HAS-BLED score | 0.942 | 0.823-1.078 | 0.381 |
| BMI | 0.962 | 0.930-0.995 | 0.025 |
| Overweight or obese | 0.816 | 0.609-1.094 | 0.174 |
| Current smoker | 1.420 | 1.031-1.957 | 0.032 |
| Hyperlipidaemia | 0.619 | 0.483-0.795 | <0.001 |
| Any comorbidity | 1.152 | 0.623-2.129 | 0.652 |
| Hypertension | 1.206 | 0.815-1.785 | 0.350 |
| Coronary artery disease | 0.922 | 0.686-1.239 | 0.590 |
| Diabetes mellitus | 0.987 | 0.735-1.325 | 0.930 |
| Heart failure | 0.942 | 0.710-1.251 | 0.681 |
| Prior stroke / TIA | 1.070 | 0.690-1.661 | 0.763 |
| Renal impairment | 0.583 | 0.356-0.956 | 0.033 |
| Obstructive sleep apnoea | 0.251 | 0.029-2.153 | 0.207 |
| Multimorbidity | 0.783 | 0.595-1.030 | 0.081 |
| OAC-naive | 1.464 | 1.132-1.893 | 0.004 |
| OAC-experienced | 0.683 | 0.528-0.883 | 0.004 |

AF- Atrial Fibrillation; CHA_2_DS_2_-VASc score, Congestive Heart Failure History, Hypertension History, Age ≥ 75 Years, Age 65 to 74 Years, Diabetes Mellitus history, Stroke History, Vascular Disease History, female sex; HAS-BLED, Hypertension, Abnormal Renal Function, Abnormal Liver Function, Stroke History, Bleeding History or Predisposition, Labile INR, Elderly > 65 Years, Drugs or Alcohol; BMI, Body Mass Index, TIA, Transient Ischemic Attack; OAC- oral anticoagulant therapy;

**Table S12. Univariate binary logistic regression of patient satisfaction at Month 12: Neutral, dissatisfied or very dissatisfied**

| **Variable** | **Odds ratio** | **95% CI** | **P value** |
| --- | --- | --- | --- |
| Age | 0.984 | 0.936-1.035 | 0.531 |
| Age under 65 | 1.552 | 0.548-4.398 | 0.408 |
| Age 65-74 | 0.809 | 0.286-2.290 | 0.690 |
| Age 75-80 | 1.228 | 0.343-4.398 | 0.752 |
| Age over 80 | 0.001 | 0.001-NR | 0.997 |
| Female sex | 1.485 | 0.535.4.128 | 0.448 |
| Newly diagnosed AF | 2.786 | 0.945-8.210 | 0.063 |
| CHA_2_DS_2_-VASc score | 1.075 | 0.778-1.486 | 0.661 |
| HAS-BLED score | 0.777 | 0.421-1.434 | 0.419 |
| BMI | 1.049 | 0.932-1.182 | 0.428 |
| Overweight or obese | 1.962 | 0.440-8.756 | 0.377 |
| Current smoker | 1.146 | 0.320-4.102 | 0.834 |
| Hyperlipidaemia | 1.558 | 0.550-4.409 | 0.404 |
| Any comorbidity | 25215524.37 | 0.001-NR | 0.998 |
| Hypertension | 27288423.20 | 0.001-NR | 0.996 |
| Coronary artery disease | 0.847 | 0.237-3.028 | 0.799 |
| Diabetes mellitus | 0.847 | 0.237-3.028 | 0.799 |
| Heart failure | 0.720 | 0.202-2.572 | 0.613 |
| Prior stroke / TIA | 0.616 | 0.137-2.774 | 0.528 |
| Renal impairment | 0.001 | 0.001-NR | 0.997 |
| Obstructive sleep apnoea | 0.001 | 0.001-NR | 0.999 |
| Multimorbidity | 0.769 | 0.260-2.269 | 0.634 |
| OAC-naive | 4.088 | 0.917-18.211 | 0.065 |
| OAC-experienced | 0.245 | 0.055-1.090 | 0.065 |

AF- Atrial Fibrillation; CHA_2_DS_2_-VASc score, Congestive Heart Failure History, Hypertension History, Age ≥ 75 Years, Age 65 to 74 Years, Diabetes Mellitus history, Stroke History, Vascular Disease History, female sex; HAS-BLED, Hypertension, Abnormal Renal Function, Abnormal Liver Function, Stroke History, Bleeding History or Predisposition, Labile INR, Elderly > 65 Years, Drugs or Alcohol; BMI, Body Mass Index, TIA, Transient Ischemic Attack; OAC- oral anticoagulant therapy;

**Table S13. Univariate linear regression of patient satisfaction at Month 3**

| **Variable** | **Coefficient B** | **SE** | **Beta** | **95% CI** | **P value** |
| --- | --- | --- | --- | --- | --- |
| Age | 0.001 | 0.002 | 0.015 | -0.003-0-005 | 0.640 |
| Age under 65 | -0.031 | -0.031 | -0.025 | -0.108-0.046 | 0.425 |
| Age 65-74 | 0.019 | 0.036 | 0.017 | -0.052-0.090 | 0.595 |
| Age 75-80 | -0.056 | -0.056 | -0.037 | -0.150-0.038 | 0.242 |
| Age over 80 | 0.136 | 0.067 | 0.063 | 0.004-0.268 | 0.043 |
| Female sex | 0.075 | 0.036 | 0.064 | 0.004-0.145 | 0.039 |
| First-diagnosed AF | 0.075 | 0.036 | 0.064 | 0.003-0.146 | 0.040 |
| CHA_2_DS_2_-VASc score | 0.005 | 0.012 | 0.014 | -0.018-0.028 | 0.658 |
| HAS-BLED score | -0.016 | 0.020 | -0.025 | -0.054-0.023 | 0.419 |
| BMI | -0.007 | 0.005 | -0.046 | -0.016-0.002 | 0.142 |
| Overweight or obese | -0.038 | 0.043 | -0.027 | -0.120-0.047 | 0.390 |
| Current smoker | 0.023 | 0.047 | 0.015 | -0.069-0.114 | 0.629 |
| Hyperlipidaemia | -0.170 | 0.036 | -0.148 | -0.240-(-0.100) | <0.001 |
| Any comorbidity | 0.097 | 0.088 | 0.035 | -0.076-0.0271 | 0.270 |
| Hypertension | -0.054 | 0.056 | -0.030 | -0.164-0.057 | 0.342 |
| Coronary artery disease | -0.017 | 0.043 | -0.012 | -0.101-0.067 | 0.697 |
| Diabetes mellitus | -0.056 | 0.043 | -0.041 | -0.140-0.028 | 0.193 |
| Heart failure | 0.017 | 0.041 | 0.013 | -0.064-0.098 | 0.680 |
| Prior stroke / TIA | 0.041 | 0.064 | 0.020 | -0.084-0.166 | 0.519 |
| Renal impairment | -0.003 | 0.068 | -0.001 | -0.136-0.131 | 0.971 |
| Obstructive sleep apnoea | -0.292 | 0.235 | -0.039 | -0.753-0.169 | 0.214 |
| Multimorbidity | -0.084 | 0.040 | -0.066 | -0.163-(-0.006) | 0.036 |
| OAC-naive | 0.075 | 0.037 | 0.064 | 0.003-0.148 | 0.041 |
| OAC-experienced | -0.075 | 0.037 | -0.064 | -0.148-(-0.003) | 0.041 |

AF- Atrial Fibrillation; CHA_2_DS_2_-VASc score, Congestive Heart Failure History, Hypertension History, Age ≥ 75 Years, Age 65 to 74 Years, Diabetes Mellitus history, Stroke History, Vascular Disease History, female sex; HAS-BLED, Hypertension, Abnormal Renal Function, Abnormal Liver Function, Stroke History, Bleeding History or Predisposition, Labile INR, Elderly > 65 Years, Drugs or Alcohol; BMI, Body Mass Index, TIA, Transient Ischemic Attack; OAC- oral anticoagulant therapy;

**Table S14. Univariate linear regression of patient satisfaction at Month 6**

| Variable | Coefficient B | SE | Beta | 95% CI | P value |
| --- | --- | --- | --- | --- | --- |
| Age | 0.001 | 0.002 | 0.025 | -0.002-0.005 | 0.427 |
| Age under 65 | -0.019 | 0.039 | -0.015 | -0.096-0.058 | 0.624 |
| Age 65-74 | -0.018 | 0.036 | -0.016 | -0.089-0.053 | 0.610 |
| Age 75-80 | 0.025 | 0.048 | 0.016 | -0.069-0.119 | 0.600 |
| Age over 80 | 0.071 | 0.067 | 0.033 | -0.061-0.203 | 0.291 |
| Female sex | 0.080 | 0.036 | 0.069 | 0.009-0.151 | 0.027 |
| First-diagnosed AF | 0.088 | 0.036 | 0.075 | 0.016-0.159 | 0.016 |
| CHA_2_DS_2_-VASc score | 0.011 | 0.012 | 0.029 | -0.012-0.034 | 0.363 |
| HAS-BLED score | 0.022 | 0.020 | 0.035 | -0.017-0.060 | 0.267 |
| BMI | -0.005 | 0.005 | -0.034 | -0.015-0.004 | 0.284 |
| Overweight or obese | 0.024 | 0.043 | 0.018 | -0.059-0.108 | 0.568 |
| Current smoker | 0.070 | 0.047 | 0.046 | -0.022-0.162 | 0.138 |
| Hyperlipidaemia | -0.096 | 0.036 | -0.083 | -0.166-(-0.025) | 0.008 |
| Any comorbidity | -0.006 | 0.089 | -0.002 | -0.180-0.168 | 0.943 |
| Hypertension | 0.072 | 0.056 | 0.040 | -0.039-0.183 | 0.203 |
| Coronary artery disease | -0.061 | 0.043 | -0.044 | -0.145-0.024 | 0.158 |
| Diabetes mellitus | 0.023 | 0.043 | 0.017 | -0.061-0.107 | 0.591 |
| Heart failure | -0.033 | 0.041 | -0.025 | -0.114-0.048 | 0.428 |
| Prior stroke / TIA | 0.013 | 0.064 | 0.006 | -0.113-0.138 | 0.845 |
| Renal impairment | -0.011 | 0.068 | -0.005 | -0.145-0.123 | 0.872 |
| Obstructive sleep apnoea | -0.352 | 0.235 | -0.047 | -0.814-0.110 | 0.135 |
| Multimorbidity | -0.055 | 0.040 | -0.043 | -0.134-0.024 | 0.171 |
| OAC-naive | 0.106 | 0.037 | 0.090 | 0.033-0.178 | 0.004 |
| OAC-experienced | -0.106 | 0.037 | -0.090 | -0.178-(.0.033) | 0.004 |

AF- Atrial Fibrillation; CHA_2_DS_2_-VASc score, Congestive Heart Failure History, Hypertension History, Age ≥ 75 Years, Age 65 to 74 Years, Diabetes Mellitus history, Stroke History, Vascular Disease History, female sex; HAS-BLED, Hypertension, Abnormal Renal Function, Abnormal Liver Function, Stroke History, Bleeding History or Predisposition, Labile INR, Elderly > 65 Years, Drugs or Alcohol; BMI, Body Mass Index, TIA, Transient Ischemic Attack; OAC- oral anticoagulant therapy;

**Table S15. Univariate linear regression of patient satisfaction at Month 12**

| **Variable** | **Coefficient B** | **SE** | **Beta** | **95% CI** | **P value** |
| --- | --- | --- | --- | --- | --- |
| Age | 0.002 | 0.002 | 0.027 | -0.002-0.005 | 0.382 |
| Age under 65 | -0.026 | 0.036 | -0.022 | -0.097-0.045 | 0.477 |
| Age 65-74 | -0.021 | 0.033 | -0.020 | -0.087-0.044 | 0.525 |
| Age 75-80 | 0.064 | 0.044 | 0.045 | -0.023-0.151 | 0.147 |
| Age over 80 | 0.023 | 0.062 | 0.012 | -0.099-0.145 | 0.713 |
| Female sex | 0.063 | 0.034 | 0.058 | -0.003-0.128 | 0.062 |
| First-diagnosed AF | 0.092 | 0.034 | 0.085 | 0.026-0.158 | 0.006 |
| CHA_2_DS_2_-VASc score | 0.010 | 0.011 | 0.030 | -0.011-0.031 | 0.341 |
| HAS-BLED score | -0.023 | 0.018 | -0.039 | -0.058-0.013 | 0.208 |
| BMI | -0.008 | 0.004 | -0.055 | -0.016-0.001 | 0.081 |
| Overweight or obese | -0.033 | 0.040 | -0.026 | -0.111-0.045 | 0.406 |
| Current smoker | 0.097 | 0.043 | 0.070 | 0.012-0.182 | 0.026 |
| Hyperlipidaemia | -0.103 | 0.033 | -0.097 | -0.168-(-0.038) | 0.002 |
| Any comorbidity | 0.066 | 0.082 | 0.025 | -0.095-0.227 | 0.419 |
| Hypertension | 0.080 | 0.052 | 0.048 | -0.022-0.182 | 0.126 |
| Coronary artery disease | -0.026 | 0.040 | -0.020 | -0.104-0.052 | 0.516 |
| Diabetes mellitus | -0.004 | 0.040 | -0.003 | -0.082-0.074 | 0.929 |
| Heart failure | -0.025 | 0.038 | -0.020 | -0.100-0.050 | 0.517 |
| Prior stroke / TIA | -0.001 | 0.059 | 0.000 | -0.117-0.115 | 0.991 |
| Renal impairment | -0.160 | 0.063 | -0.079 | -0.283-(-0.037) | 0.011 |
| Obstructive sleep apnoea | -0.308 | 0.218 | -0.044 | -0.735-0.119 | 0.158 |
| Multimorbidity | -0.067 | 0.37 | -0.057 | -0.140-0.005 | 0.070 |
| OAC-naive | 0.122 | 0.034 | 0.111 | 0.055-0.188 | <0.001 |
| OAC-experienced | -0.122 | 0.034 | -0.111 | -0.188-(-0.055) | <0.001 |

AF- Atrial Fibrillation; CHA_2_DS_2_-VASc score, Congestive Heart Failure History, Hypertension History, Age ≥ 75 Years, Age 65 to 74 Years, Diabetes Mellitus history, Stroke History, Vascular Disease History, female sex; HAS-BLED, Hypertension, Abnormal Renal Function, Abnormal Liver Function, Stroke History, Bleeding History or Predisposition, Labile INR, Elderly > 65 Years, Drugs or Alcohol; BMI, Body Mass Index, TIA, Transient Ischemic Attack; OAC- oral anticoagulant therapy;

**Table S16.** **Multivariate linear regression analyses of patient self-reported satisfaction with DOAC therapy at the 3-, 6- and 12-month follow-up visit.**

|  | | **Multivariate linear regression^a^** | | | | | | | |
| --- | --- | --- | --- | --- | --- | --- | --- | --- | --- |
| **M3** | |  | | | | |  | |  |
| *Variable* | | **B** | | **SE** | | **Beta** | **95% CI** | | **P value** |
| **Hyperlipidaemia** | | -0.181 | | 0.036 | | -0.158 | -0.252-(-0.110) | | <0.001 |
| OAC-naive | |  | |  | |  |  | |  |
| OAC-experienced | |  | |  | |  |  | |  |
| **M6** | |  | |  | |  |  | |  |
| **Female sex** | | 0.077 | | 0.036 | | 0.067 | 0.006-0.148 | | 0.032 |
| **Hyperlipidaemia** | | -0.094 | | 0.037 | | -0.082 | -0.165-(-0.022) | | 0.010 |
| **OAC-naive** | | 0.102 | | 0.037 | | 0.086 | 0.029-0.175 | | 0.006 |
| **OAC-experienced** | |  | |  | |  |  | |  |
| **M12** |  | |  | |  | | |  | |
| **Female sex** | | 0.085 | | 0.036 | | 0.073 | 0.014-0.156 | | 0.019 |
| **Hyperlipidaemia** | | -0.095 | | 0.037 | | -0.083 | -0.167-(-0.024) | | 0.009 |
| **OAC-naive** | | 0.099 | | 0.037 | | 0.083 | 0.026-0.172 | | 0.008 |
| **OAC-experienced** | |  | |  | |  |  | |  |

^a^ adjusted for Age, Sex, CHA2DS2-VASc score, HAS-BLED score, Multimorbidity, Comorbidity, Renal Impairment, Hyperlipidaemia and First-diagnosed AF

M-month, OAC- oral anticoagulant therapy
